# Metabolic-BMI phenotypes as nutritional risk indicators for osteoarthritis: evidence from a prospective cohort of UK adults

**DOI:** 10.3389/fnut.2025.1722731

**Published:** 2025-12-11

**Authors:** Lei Bao, Lincheng Duan

**Affiliations:** 1 Traditional Chinese Medicine Prevention and Treatment Center, Chengdu Integrated TCM and Western Medicine Hospital, Chengdu, China; 2 Acupuncture and Tuina School, Chengdu University of Traditional Chinese Medicine, Chengdu, China

**Keywords:** osteoarthritis, obesity, metabolic phenotype, English Longitudinal Study of Ageing (ELSA), middle-aged and older adults

## Abstract

**Background:**

Metabolic health status has emerged as a crucial nutritional and physiological indicator, reflecting the complex interplay between dietary intake, energy metabolism, and chronic disease risk. Obesity is a well-known risk factor for osteoarthritis (OA), yet substantial metabolic heterogeneity exists among individuals with obesity. Understanding how metabolic variability modifies the obesity-OA relationship can provide valuable insights into nutrition-related mechanisms of musculoskeletal health. This study aimed to examine the association between metabolic-BMI phenotypes and incident OA among middle-aged and older adults (MAOA) in the UK.

**Methods:**

The ELSA provided the data. Metabolically healthy normal weight (MHNW), metabolically healthy overweight/obesity (MHOO), metabolically unhealthy normal weight (MUNW), as well as metabolically unhealthy overweight/obesity (MUOO) were the four metabolic-BMI phenotypes into which the participants were divided. The relationships between these phenotypes and incident OA were estimated via Cox proportional hazards models, which controlled for clinical variables, lifestyle choices, and sociodemographic traits.

**Results:**

During a median follow-up of 10 years, 673 new OA cases were identified. Compared with the MHNW group, participants with MHOO (HR = 1.54, 95% CI: 1.19–2.01, *p* = 0.001) as well as MUOO (HR = 1.90, 95% CI: 1.46–2.47, *p* < 0.001) had significantly higher risks of developing OA, while no remarkable association was seen for the MUNW group (HR = 1.00, 95% CI: 0.68–1.46, *p* = 0.99). In the longitudinal analysis, individuals who remained in MUNW, MHOO, or MUOO categories—or transitioned from MHNW to MUOO, or from metabolically healthy to unhealthy states within the obese group—showed markedly increased risks of OA onset.

**Conclusion:**

Metabolic status modified—but did not eliminate—the association between obesity and osteoarthritis risk. These findings highlight substantial heterogeneity within obesity phenotypes and suggest that incorporating metabolic health with BMI may improve identification of individuals at elevated OA risk.

## 1 Introduction

Osteoarthritis (OA) is one of the leading causes of pain and disability among middle-aged and older adults worldwide. Its disease burden continues to rise in the context of global population ageing, posing substantial public health and socioeconomic challenges (1). Obesity is widely recognized as a major risk factor for OA. Beyond mechanical loading on weight-bearing joints, obesity may also contribute to OA development and progression through metabolic pathways involving chronic low-grade inflammation, adipokine dysregulation, and disturbances in the cartilage-subchondral bone homeostasis (2–4). However, obesity is not a metabolically homogeneous condition. Some individuals with overweight or obesity remain metabolically healthy, whereas others with normal body weight may exhibit metabolic abnormalities. This phenomenon of “metabolic heterogeneity” suggests BMI alone may be insufficient to capture OA risk, and that combined metabolic health-BMI phenotypes may provide a more accurate characterization of inter-individual differences in disease susceptibility (5–8).

OA is increasingly understood as a condition shaped not only by mechanical loading but also by systemic nutritional-metabolic processes (9). A growing body of literature has demonstrated that metabolic abnormalities—such as dyslipidemia, hypertension, central adiposity, and impaired glucose regulation—are associated with higher risks of OA across both weight-bearing and non-weight-bearing joints (10–12). These metabolic disturbances represent key nutritional and physiological indicators, suggesting that metabolic health may influence joint vulnerability independently of body mass.

Body mass index (BMI), although widely used, captures only one dimension of nutritional status and does not adequately reflect metabolic health. Recent large-scale analyses from the UK Biobank (13, 14) indicate that metabolic syndrome and its components are prospectively associated with elevated OA risk independent of BMI, underscoring the need to consider metabolic health alongside adiposity in OA research. Moreover, recent reviews have highlighted that obesity-related inflammatory responses and adipokine signaling may affect the joint microenvironment through multiple mechanisms, thereby accelerating joint degeneration (4, 15, 16). However, most existing studies rely on cross-sectional data or a single time-point assessment of metabolic status, limiting understanding of whether “metabolically healthy overweight/obesity” (MHOO) is a stable phenotype or a transient state preceding metabolic deterioration. Few studies have examined dynamic transitions in metabolic phenotypes over time, despite accumulating evidence that individuals classified as MHOO frequently progress to metabolically unhealthy overweight/obesity (MUOO), which may help explain the heterogeneity in long-term OA susceptibility observed in cohort studies (17–20). Addressing this gap, the present study specifically investigates both baseline metabolic-BMI phenotypes and their longitudinal transitions in relation to incident OA.

The English Longitudinal Study of Ageing (ELSA) provides repeated assessments of nutritional-metabolic indicators, behavioral factors, and clinical conditions in a nationally representative cohort of middle-aged and older adults in the UK (21). This offers a unique opportunity to investigate both the static profile of metabolic-BMI phenotypes—namely metabolically healthy normal weight (MHNW), MHOO, metabolically unhealthy normal weight (MUNW), and MUOO—and their dynamic transitions over time. Therefore, this study aimed to: (1) examine the associations between baseline metabolic-BMI phenotypes and incident OA; and (2) evaluate whether transitions in metabolic-BMI phenotypes during follow-up were associated with subsequent OA risk.

## 2 Methods

### 2.1 Study design and population

The study was a prospective cohort analysis based on data from the ELSA, which is a nationally representative cohort that has been ongoing in England since 2002, continuously tracking the health status, socioeconomic characteristics, as well as lifestyle behaviors of community-dwelling adults aged 50 years and older (21).

#### 2.1.1 Baseline and follow-up definition

**Figure 1:**
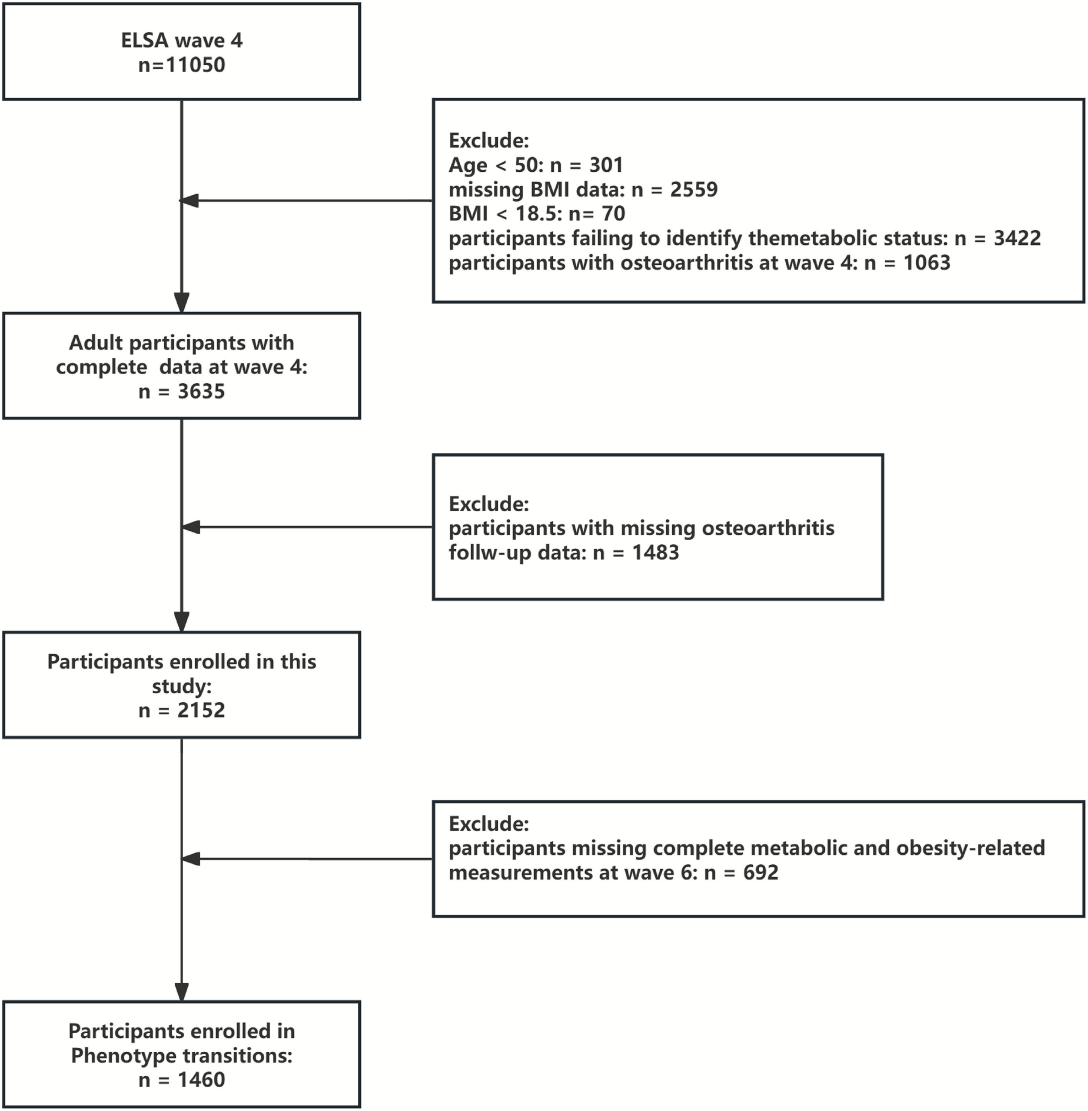
Research flowchart.

Wave 4 (2008–2009) was selected as the baseline because it was the first ELSA wave with complete and standardized measurements required to define combined metabolic-BMI phenotypes, including metabolic biomarkers, blood pressure, waist circumference, and body weight. Participants were followed from wave 4 through wave 9 (2018–2019). Individuals with a prior diagnosis of OA at baseline were excluded. After applying the inclusion and exclusion criteria, a total of 2,152 adults aged ≥50 years were included in the baseline analytic sample (Figure 1).

#### 2.1.2 Phenotype transition assessment

Phenotype transitions were examined between wave 4 and wave 6. Wave 6 (2012–2013) was chosen because it was the next wave that collected all essential metabolic and anthropometric variables required for reclassifying metabolic-BMI phenotypes; waves 5 and 7 did not contain complete metabolic data and were therefore unsuitable for transition analyses. Of the 2,152 participants included at baseline, 1,460 had complete metabolic and obesity-related measurements at wave 6 and sufficient follow-up information and were therefore included in the phenotype transition analysis (Figure 1).

### 2.2 Variable definitions and measurements

#### 2.2.1 Obesity and metabolic status

BMI was used to measure obesity. The BMIs of 18.5–24.9 kg/m^2^, 25.0–29.9 kg/m^2^, as well as ≥30.0 kg/m^2^ were used to describe normal weight, overweight, as well as obesity.

Four metabolic markers were used to define metabolic state. Individuals were categorized as metabolically unwell if they met at least two of the following requirements (22, 23): Elevated blood pressure: using antihypertensive medication at the moment, or having a systolic blood pressure of 130 mmHg or a diastolic blood pressure of 85 mmHg; Glycated hemoglobin (HbA1c) ≥6.0%, fasting blood glucose ≥5.6 mmol/L, or current usage of an antidiabetic drug are indicators of Impaired glucose regulation; Elevated triglycerides (TG): TG ≥1.7 mmol/L or using a lipid-lowering drug at the moment; Reduced high-density lipoprotein cholesterol (HDL-C): HDL-C <1.03 mmol/L for males or <1.29 mmol/L for women, or the use of lipid-lowering drugs at the time of the measurement.

The four phenotypes of MHNW, MUNW, MHOO, as well as MUOO were determined by combining BMI and metabolic state. These phenotypes were identified at both baseline and the second follow-up (approximately 4 years later). Metabolic transitions were defined according to changes in BMI-metabolic phenotypes between these two time points, categorized as either stable or transitioned phenotypes.

#### 2.2.2 OA outcome

The primary outcome was incident OA during follow-up. OA diagnosis was determined using self-reported responses to the ELSA question “Has a doctor ever told you that you have osteoarthritis?” and was confirmed across subsequent survey waves. This self-reported approach has been validated in multiple ELSA-based studies and shown to be consistent with medical records (24, 25).

### 2.3 Potential confounders

To minimize confounding bias, several covariates were included in the analysis, encompassing:

Demographic characteristics: age, sex, ethnicity, education, and marital status.
Lifestyle factors: smoking status (yes/no), drinking frequency (≥1 time/week vs. <1 time/week), and physical activity level (high/low).
Health status: presence of chronic conditions like hypertension, diabetes, as well as dyslipidemia.

Missing covariate data were imputed via multiple imputation by chained equations combined with a random forest algorithm. Details of missingness are displayed in Supplementary Table S1.

### 2.4 Statistical analysis

Categorical data were represented via frequency as well as percentage, whereas continuous variables were represented by mean ± SD or median. Using chi-square tests for categorical variables as well as *t*-tests or Kruskal–Wallis tests for continuous variables, group comparisons were carried out.

The Kaplan–Meier technique was leveraged to create cumulative incidence curves for OA, and log-rank tests were leveraged to evaluate group differences. Cox regression models were leveraged to assess the relationships of metabolic-BMI phenotypes with incident OA. The findings were displayed as HRs and 95% CIs. We evaluated the assumptions underlying the Cox proportional hazards models. Proportional hazard assumption was examined using Schoenfeld residual methods. The following gradual adjustments were made to the models: Model 1 is unadjusted, Model 2 is demographically adjusted, and Model 3 is further adjusted for comorbid conditions and lifestyle.

Subgroup analyses stratified by age, sex, education, marital status, smoking, drinking, as well as physical activity were carried out in order to evaluate robustness. Two sensitivity analyses were undertaken to assess the robustness of the main findings. First, multivariable logistic regression models were fitted to re-evaluate the associations between baseline metabolic phenotypes and incident OA, as an alternative to the Cox proportional hazards models. Second, to minimize potential reverse causation, we repeated the analyses after excluding participants who developed OA during the first follow-up wave.

R software was used for all analyses (version 4.3.1). Statistical significance was defined as two-sided *p*-values less than 0.05.

## 3 Results

### 3.1 Baseline characteristics

A total of 2,152 eligible participants were included in the analysis, with a mean age of 62.76 years; 49.02% were male, 97.72% were White, and 71.61% were married. Among the four metabolic-BMI phenotypes, the MUNW group had the smallest proportion (*n* = 195). Over a median follow-up period of 120 months, 673 incident cases of OA were identified. The incidence rate was 31.27 per 1,000 person-years. Significant differences were observed among the four phenotype groups in terms of age, sex, lifestyle behaviors, and prevalence of chronic diseases (Table 1).

**Table 1:** Baseline characteristics of study populations across BMI-metabolic phenotypes.

Variable	Levels	Overall	MHNW	MHOO	MUNW	MUOO	*p*-value
		*N* = 2,152	*N* = 383	*N* = 628	*N* = 195	*N* = 946	
Demographic characteristics							
Age, mean (SD)		62.76 (7.16)	60.93 (6.60)	61.60 (6.76)	64.90 (6.79)	63.82 (7.42)	**<0.001**
Follow-up time (months), mean (SD)		103.14 (32.67)	109.97 (25.65)	105.11 (31.04)	105.77 (30.94)	98.52 (35.81)	**<0.001**
Sex, *n* (p%)							**<0.001**
	Female	1097.00 (50.98%)	225.00 (58.75%)	324.00 (51.59%)	114.00 (58.46%)	434.00 (45.88%)	
	Male	1055.00 (49.02%)	158.00 (41.25%)	304.00 (48.41%)	81.00 (41.54%)	512.00 (54.12%)	
Race, *n* (p%)							0.066
	Non-White	49.00 (2.28%)	6.00 (1.57%)	8.00 (1.27%)	7.00 (3.59%)	28.00 (2.96%)	
	White	2103.00 (97.72%)	377.00 (98.43%)	620.00 (98.73%)	188.00 (96.41%)	918.00 (97.04%)	
Marital status, *n* (p%)							**0.002**
	Married	1541.00 (71.61%)	257.00 (67.10%)	481.00 (76.59%)	129.00 (66.15%)	674.00 (71.25%)	
	Others	611.00 (28.39%)	126.00 (32.90%)	147.00 (23.41%)	66.00 (33.85%)	272.00 (28.75%)	
Education, *n* (p%)							**<0.001**
	Below high school	569.00 (26.44%)	66.00 (17.23%)	135.00 (21.50%)	61.00 (31.28%)	307.00 (32.45%)	
	College or above	1050.00 (48.79%)	212.00 (55.35%)	343.00 (54.62%)	86.00 (44.10%)	409.00 (43.23%)	
	High school	533.00 (24.77%)	105.00 (27.42%)	150.00 (23.89%)	48.00 (24.62%)	230.00 (24.31%)	
Income (quintiles), *n* (p%)							0.516
	Q1	432.00 (20.07%)	75.00 (19.58%)	128.00 (20.38%)	33.00 (16.92%)	196.00 (20.72%)	
	Q2	429.00 (19.93%)	64.00 (16.71%)	120.00 (19.11%)	50.00 (25.64%)	195.00 (20.61%)	
	Q3	433.00 (20.12%)	86.00 (22.45%)	125.00 (19.90%)	40.00 (20.51%)	182.00 (19.24%)	
	Q4	427.00 (19.84%)	84.00 (21.93%)	119.00 (18.95%)	34.00 (17.44%)	190.00 (20.08%)	
	Q5	431.00 (20.03%)	74.00 (19.32%)	136.00 (21.66%)	38.00 (19.49%)	183.00 (19.34%)	
Lifestyle factors							
Smoke, *n* (p%)							**<0.001**
	No	1908.00 (88.66%)	327.00 (85.38%)	580.00 (92.36%)	155.00 (79.49%)	846.00 (89.43%)	
	Yes	244.00 (11.34%)	56.00 (14.62%)	48.00 (7.64%)	40.00 (20.51%)	100.00 (10.57%)	
Drink, *n* (p%)							**0.001**
	<1/week	720.00 (33.46%)	104.00 (27.15%)	196.00 (31.21%)	64.00 (32.82%)	356.00 (37.63%)	
	≥1/week	1432.00 (66.54%)	279.00 (72.85%)	432.00 (68.79%)	131.00 (67.18%)	590.00 (62.37%)	
Physical activity, *n* (p%)							**<0.001**
	High	1877.00 (87.22%)	348.00 (90.86%)	573.00 (91.24%)	171.00 (87.69%)	785.00 (82.98%)	
	Low	275.00 (12.78%)	35.00 (9.14%)	55.00 (8.76%)	24.00 (12.31%)	161.00 (17.02%)	
Health status							
Depression, *n* (p%)							0.087
	No	1962.00 (91.17%)	357.00 (93.21%)	581.00 (92.52%)	173.00 (88.72%)	851.00 (89.96%)	
	Yes	190.00 (8.83%)	26.00 (6.79%)	47.00 (7.48%)	22.00 (11.28%)	95.00 (10.04%)	
Diabetes, *n* (p%)							**<0.001**
	No	1864.00 (86.62%)	376.00 (98.17%)	619.00 (98.57%)	165.00 (84.62%)	704.00 (74.42%)	
	Yes	288.00 (13.38%)	7.00 (1.83%)	9.00 (1.43%)	30.00 (15.38%)	242.00 (25.58%)	
Hyperlipidemia, *n* (p%)							**<0.001**
	No	1525.00 (70.86%)	321.00 (83.81%)	541.00 (86.15%)	103.00 (52.82%)	560.00 (59.20%)	
	Yes	627.00 (29.14%)	62.00 (16.19%)	87.00 (13.85%)	92.00 (47.18%)	386.00 (40.80%)	
Hypertension, *n* (p%)							**<0.001**
	No	1129.00 (52.46%)	294.00 (76.76%)	433.00 (68.95%)	89.00 (45.64%)	313.00 (33.09%)	
	Yes	1023.00 (47.54%)	89.00 (23.24%)	195.00 (31.05%)	106.00 (54.36%)	633.00 (66.91%)	
Incidence of OA, *n* (p%)							**<0.001**
	No	1479.00 (68.73%)	303.00 (79.11%)	436.00 (69.43%)	150.00 (76.92%)	590.00 (62.37%)	
	Yes	673.00 (31.27%)	80.00 (20.89%)	192.00 (30.57%)	45.00 (23.08%)	356.00 (37.63%)	

The bolded values indicate that the *p*-values are less than 0.05.

### 3.2 Association between baseline metabolic**-**BMI phenotypes and OA risk

Cox proportional hazards models (Table 2) further showed that in both unadjusted and fully adjusted models, obesity and metabolic unhealthiness were related to a remarkably higher risk of incident OA. Compared with participants classified as MHNW, those with MHOO and MUOO showed 54% (HR = 1.54, 95% CI: 1.18–2.00, *p* = 0.001) as well as 89% (HR = 1.89, 95% CI: 1.45–2.46, *p* < 0.001) higher risks of OA, respectively. No significant association was found for the MUNW phenotype (HR = 1.00, 95% CI: 0.68–1.46, *p* = 0.99). The proportional hazards (PH) assumption was met. The global Schoenfeld residuals test was non-significant (*p* > 0.05).

**Table 2:** Correlation between BMI-metabolic phenotypes and the risk of OA.

Variables	Model 1		Model 2		Model 3	
	HR (95% CI)	*p*	HR (95% CI)	*p*	HR (95% CI)	*p*
Metabolic-BMI phenotypes						
MHNW	1.00 (Reference)		1.00 (Reference)		1.00 (Reference)	
MUNW	1.16 (0.81–1.67)	0.426	1.05 (0.72–1.52)	0.803	1.00 (0.68–1.46)	0.992
MHOO	1.53 (1.18–1.99)	**0.001**	1.57 (1.20–2.03)	**<0.001**	1.54 (1.18–2.00)	**0.001**
MUOO	2.03 (1.59–2.59)	**<0.001**	2.02 (1.58–2.59)	**<0.001**	1.89 (1.45–2.46)	**<0.001**

HR, hazard ratio; CI, confidence interval.

Model 1: Crude.

Model 2: Adjust: Age, sex, marital status, race, education, income.

Model 3: Adjust: Age, sex, marital status, race, education, income, smoke, drink, physical activity, depression, diabetes, hyperlipidemia, hypertension.

The bolded values indicate that the *p*-values are less than 0.05.

**Figure 2:**
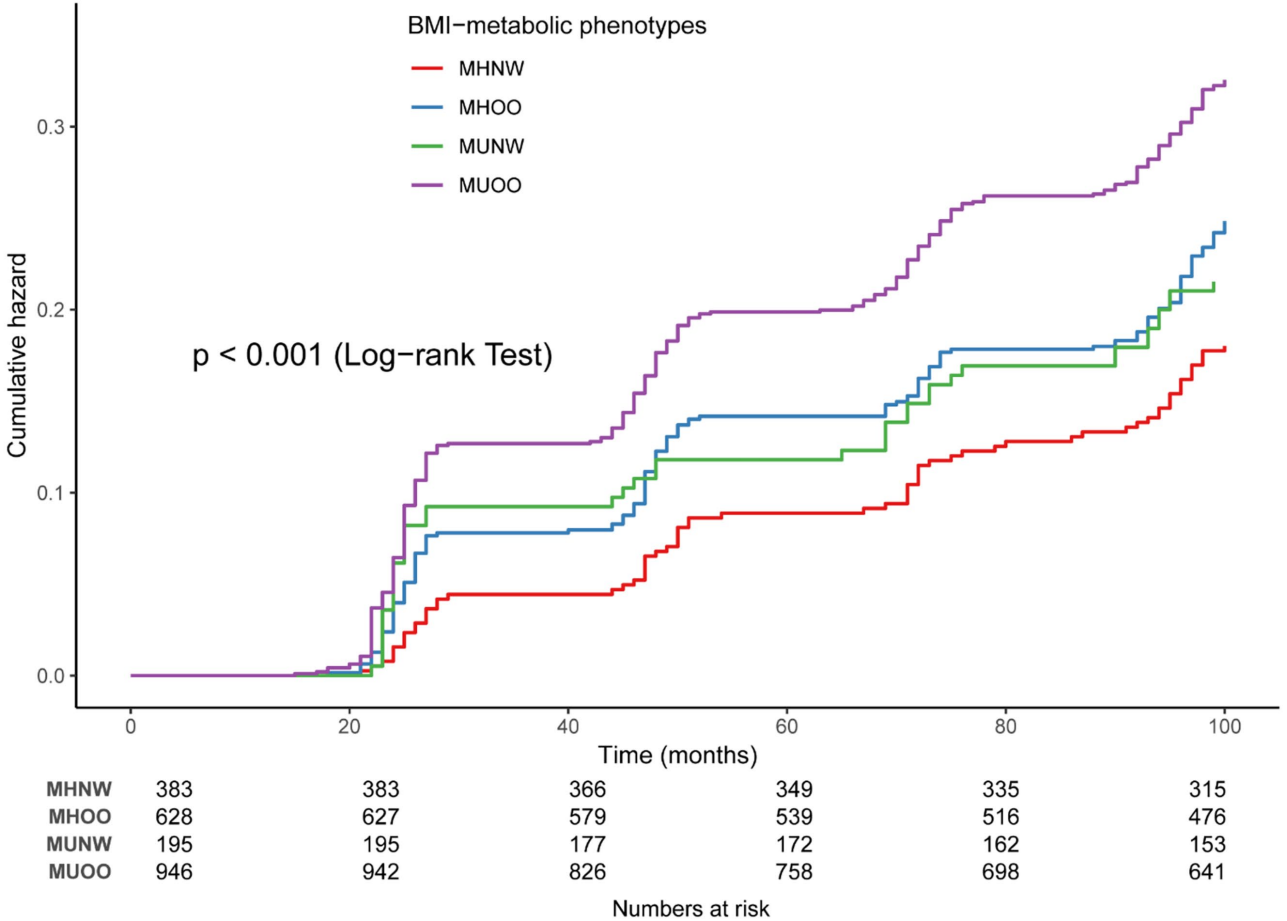
Kaplan–Meier curves for the cumulative incidence of OA.

The Kaplan–Meier survival curves demonstrated a remarkable difference in cumulative OA incidence across the metabolic-BMI phenotypes (log-rank test, *p* < 0.001) (Figure 2).

### 3.3 Association between phenotypic transitions and OA risk

Considering the potential dynamic nature of BMI and metabolic status, we further explored the associations between phenotype transitions from wave 4 to wave 6 and the subsequent risk of OA (Table 3). Using participants who remained metabolically healthy and of normal weight as the reference group, those who persistently exhibited MHOO or MUOO had significantly increased OA risks, with HRs of 2.26 (95% CI: 1.14–4.48, *p* = 0.020) as well as 2.60 (95% CI: 1.39–4.88, *p* = 0.003).

**Table 3:** Association between phenotypic transitions and OA risk.

Variables	Model 1		Model 2		Model 3	
	HR (95% CI)	*p*	HR (95% CI)	*p*	HR (95% CI)	*p*
Change						
MHNW → MHNW	1.00 (Reference)		1.00 (Reference)		1.00 (Reference)	
MHNW → MHOO	1.82 (0.24–14.01)	0.564	1.53 (0.20–11.81)	0.684	1.41 (0.18–11.02)	0.743
MHNW → MUNW	1.26 (0.57–2.75)	0.570	1.26 (0.57–2.76)	0.568	1.17 (0.53–2.58)	0.705
MHNW → MUOO	3.10 (1.27–7.59)	**0.013**	3.48 (1.42–8.54)	**0.007**	3.27 (1.33–8.05)	**0.010**
MHOO → MHNW	2.73 (0.88–8.47)	0.082	2.96 (0.94–9.26)	0.063	2.97 (0.95–9.30)	0.062
MHOO → MHOO	2.19 (1.11–4.32)	**0.024**	2.31 (1.17–4.57)	**0.016**	2.26 (1.14–4.48)	**0.020**
MHOO → MUNW	3.71 (1.20–11.50)	**0.023**	3.89 (1.24–12.19)	**0.020**	3.56 (1.12–11.23)	**0.031**
MHOO → MUOO	2.28 (1.23–4.24)	**0.009**	2.39 (1.28–4.46)	**0.006**	2.20 (1.17–4.14)	**0.014**
MUNW → MHNW	0.00 (0.00–Inf)	0.989	0.00 (0.00–Inf)	0.989	0.00 (0.00–Inf)	0.990
MUNW → MUNW	1.11 (0.47–2.63)	0.818	1.07 (0.45–2.56)	0.876	0.96 (0.39–2.33)	0.927
MUNW → MUOO	2.85 (1.12–7.24)	**0.028**	2.91 (1.13–7.47)	**0.026**	2.71 (1.03–7.08)	**0.043**
MUOO → MHNW	5.37 (0.70–41.34)	0.106	5.63 (0.72–43.74)	0.099	3.56 (0.45–28.46)	0.231
MUOO → MHOO	1.64 (0.62–4.37)	0.322	1.68 (0.62–4.50)	0.306	1.74 (0.65–4.69)	0.273
MUOO → MUNW	1.67 (0.54–5.17)	0.376	1.53 (0.49–4.79)	0.461	1.35 (0.42–4.34)	0.609
MUOO → MUOO	2.72 (1.51–4.91)	**<0.001**	2.87 (1.57–5.21)	**<0.001**	2.60 (1.39–4.88)	**0.003**

HR, hazard ratio; CI, confidence interval.

Model 1: Crude.

Model 2: Adjust: Age, sex, marital status, race, education, income.

Model 3: Adjust: Age, sex, marital status, race, education, income, smoke, drink, physical activity, depression, diabetes, hyperlipidemia, hypertension.

The bolded values indicate that the *p*-values are less than 0.05.

Participants whose metabolic profiles deteriorated from MHNW to MUOO also showed a markedly elevated risk (HR = 3.27, 95% CI: 1.33–8.05, *p* = 0.010), as did those transitioning from MHOO to MUOO (HR = 2.20, 95% CI: 1.17–4.14, *p* = 0.014). Similarly, individuals shifting from MUNW to MUOO had an higher risk of OA (HR = 2.71, 95% CI: 1.03–7.08, *p* = 0.043).

Overall, participants with persistent obesity or those whose metabolic condition worsened over time were at the greatest risk of developing OA, suggesting that metabolic deterioration in the presence of obesity may be a key determinant in OA pathogenesis as well as progression.

### 3.4 Subgroup analyses

**Figure 3:**
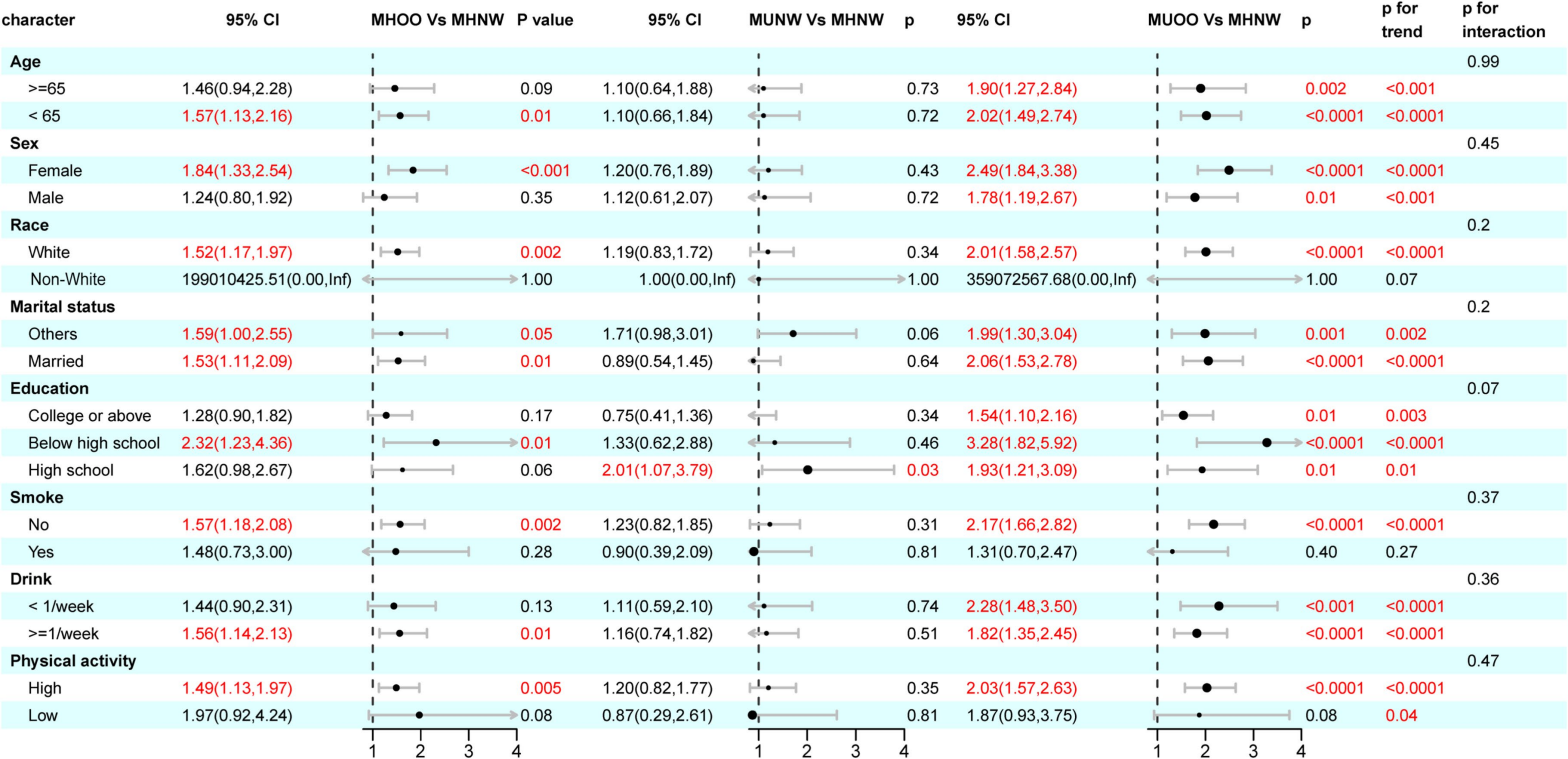
The association between BMI-metabolic phenotypes and OA in different subgroups.

Subgroup analyses (Figure 3) showed consistent positive associations between metabolic-BMI phenotypes and OA risk across various subgroups stratified by age (<65 vs. ≥65 years), sex, educational attainment, marital status, smoking, drinking frequency, as well as physical activity levels. No significant interactions were observed between phenotypes and subgroup factors (all *p*-interaction >0.05), indicating good robustness and generalizability of the findings.

### 3.5 Sensitivity analyses

Two sensitivity analyses were performed to test the robustness of the main results. (1) Multivariable logistic regression models were used to reassess the relationship between baseline phenotypes and OA risk, yielding results consistent with those from the Cox models (Supplementary Table S2). (2) After excluding incident OA cases identified in the first follow-up wave to minimize potential reverse causation, the associations and effect sizes remained largely unchanged (Supplementary Table S3). Overall, the sensitivity analyses confirmed the robustness and stability of the primary findings.

## 4 Discussion

In this prospective cohort study of adults aged ≥50 years in the United Kingdom, we observed that both MHOO and MUOO phenotypes were statistically associated with a higher likelihood of incident OA compared with MHNW individuals, whereas no significant association was identified for MUNW. These findings suggest that the association between obesity and OA risk is not determined by body weight alone but may also reflect underlying metabolic health differences. Accordingly, reliance on BMI alone may overlook risk heterogeneity, whereas combining metabolic health and BMI—and considering their longitudinal changes—may improve the identification of individuals at elevated OA risk. However, given the observational nature of the study, these results should not be interpreted as causal.

Our results are consistent with prior evidence linking obesity to OA and further extend the understanding of this relationship by incorporating metabolic heterogeneity and temporal dynamics. Obesity contributes to OA development and progression through both mechanical loading and metabolic inflammation pathways (2, 4). The large-scale UK Biobank study demonstrated MetS was related with a higher risk of OA (overall HR ≈ 1.15), with central obesity showing a particularly strong effect (HR ≈ 1.58) (13). Similarly, we observed a graded association in which OA risk increased with metabolic deterioration (MUOO > MHOO > MHNW), supporting the independent role of metabolic dysfunction in the obesity-OA axis. Metabolic abnormalities may accelerate cartilage degeneration and subchondral bone remodeling through systemic inflammation, adipokine imbalance, insulin resistance, and oxidative stress, thereby amplifying the mechanical burden of obesity.

Unlike most previous studies that defined metabolic phenotypes at a single time point, our analysis highlights the dynamic nature of metabolic status. Epidemiological evidence has shown metabolic health is often transient—approximately 40% of metabolically healthy overweight or obese individuals transition to a metabolically unhealthy state during follow-up (26). Consistent with this, our results revealed that participants who remained in the MUOO state or transitioned from MHOO to MUOO had a substantially increased risk of developing OA. Recent longitudinal evidence suggests that over 70% of MHOO individuals convert to MUOO status across multiple follow-up assessments (27). Collectively, these findings suggest “metabolically healthy obesity” is not a stable or benign condition but rather a transitional state influenced by lifestyle and metabolic stress. The shift in adipokine profiles and the elevation of inflammatory thresholds may underlie the progressive increase in OA risk observed among obese individuals over time.

Emerging research has expanded the obesity-OA relationship into a broader metabolic-immune-ageing framework. White adipose tissue and the infrapatellar fat pad are now recognized as active sources of local pro-inflammatory cytokines as well as adipokines, including TNF-α, IL-6, leptin, as well as resistin (28–30). These mediators amplify synovial inflammation, upregulate cartilage-degrading enzymes (MMP-13, ADAMTS-5), and disrupt cartilage-subchondral bone homeostasis, directly contributing to OA pathogenesis. The NLRP3 inflammasome, repeatedly observed to be activated in OA, promotes caspase-1–dependent maturation of IL-1β as well as IL-18, triggers pyroptosis, and interacts with the senescence-associated secretory phenotype, perpetuating a cycle of metabolic dysregulation and joint inflammation (31–33). Moreover, the AGEs-RAGE signaling axis, activated under hyperglycemic and insulin-resistant conditions, induces oxidative stress and NF-κB pathway activation, accelerating cartilage matrix glycation, stiffening, and loss of reparative capacity (34, 35). Collectively, these mechanisms constitute a biological basis linking obesity, metabolic dysfunction, and joint degeneration, aligning with the phenotypic associations observed in our study.

The findings of the research have important clinical and public health implications. OA risk assessment should move beyond reliance on BMI alone toward a combined framework incorporating both metabolic health and body weight status, with an emphasis on monitoring phenotypic transitions over time. Individuals persistently classified as MUOO, those transitioning from MHOO to MUOO, and metabolically unhealthy normal-weight individuals should be prioritized for OA prevention. Interventions should shift from focusing solely on weight reduction to a dual strategy of weight management and metabolic optimization, emphasizing blood pressure, glucose, lipid, as well as waist circumference control, as well as incorporating resistance training and joint-friendly aerobic exercise to improve metabolic health and joint function. International guidelines likewise highlight exercise, patient education, and weight management as core OA interventions, while metabolic optimization may further enhance their effectiveness (36).

Several limitations should be acknowledged. First, OA diagnosis was based on self-reported or physician-reported data rather than standardized imaging criteria, which may introduce nondifferential misclassification and bias estimates toward the null (37). Second, there is no universally accepted definition of metabolic health, and variations in threshold criteria may affect phenotype classification and comparability across studies (5, 38) Third, BMI cannot fully capture body composition, fat distribution, or muscle mass (e.g., visceral adiposity or sarcopenia) (39). Moreover, phenotype updates based on biennial survey waves may not precisely reflect transition timing or cumulative exposure (40). In addition, inherent structural constraints of the ELSA dataset should be noted. Key variables—including waist circumference, blood biomarkers (e.g., CRP), and medication histories—were available only in selected waves or among participants who consented to blood sampling, leading to substantial missingness and potential selection bias. Fasting insulin was not measured, preventing calculation of HOMA-IR. Moreover, detailed data on occupational mechanical loading and dose-specific longitudinal medication use (e.g., NSAIDs, analgesics, HRT, metabolic agents) were not comprehensively captured. Incorporating these variables would have substantially reduced the analytic sample and may have introduced further bias. Although multiple covariates were adjusted for, residual confounding from factors such as occupational loading, diet quality, medication use (analgesics or metabolic agents), and reverse causality due to reduced activity in early OA cannot be entirely ruled out (41). Lastly, the study population comprised adults aged ≥50 years in the UK, and generalizability to other ethnic or healthcare settings may be limited.

Future research should incorporate richer assessments of biomechanical exposure, including detailed occupational joint-load metrics and lifetime physical workload histories. Linkage to external prescription databases would allow more accurate capture of medication use, including NSAIDs, metabolic agents, and hormone therapy, with dose and duration information. In addition, the application of advanced causal inference methods—such as marginal structural models, negative control analyses, and quantitative bias analysis—may help reduce residual confounding and strengthen causal interpretation in longitudinal ageing cohorts.

## 5 Conclusion

This study identifies associations, not causation, between time-updated metabolic phenotypes, including transitions—and incident OA. Phenotypes marked by metabolic unhealth were associated with higher OA risk across the weight spectrum, whereas reliance on BMI alone risks obscuring central adiposity and mischaracterizing metabolic status. Future studies with imaging-based OA endpoints, direct body composition, inflammatory biomarkers, and higher-frequency metabolic profiling are needed to delineate mechanisms and to test whether improving metabolic health modifies OA risk.

## Data Availability

The original contributions presented in the study are included in the article/Supplementary material, further inquiries can be directed to the corresponding author.
