# Supplementary material for: Metabolic-BMI phenotypes as nutritional risk indicators for osteoarthritis: evidence from a prospective cohort of UK adults
Source: Front Nutr. 2025 Dec 11;12:1722731. doi: 10.3389/fnut.2025.1722731 (PMC12739954; doi:10.3389/fnut.2025.1722731)
Supplement: Supplementary file 1 [file Table_1.DOCX]

Supplementary Material

**Supplemental Tables S1.** The numbers (percentages) of participants with missing covariates.

| **Covariate** | **Number** | **Percentage (%)** |
| --- | --- | --- |
| depression | 3 | 0.1 |
| Smoke | 15 | 0.7 |
| Drink | 148 | 6.9 |
| Education | 164 | 7.6 |
| Race | 1 | 0.03 |

**Supplemental Tables S2**. Logistic Regression Estimates of the Association between BMI-metabolic phenotypes and the Risk of incident OA.

| Variables | Model1 | |  | Model2 | |  | Model3 | |
| --- | --- | --- | --- | --- | --- | --- | --- | --- |
|  | OR (95%CI) | *P* |  | OR (95%CI) | *P* |  | OR (95%CI) | *P* |
| MHO |  |  |  |  |  |  |  |  |
| MHNW | 1.00 (Reference) |  |  | 1.00 (Reference) |  |  | 1.00 (Reference) |  |
| MUNW | 1.14 (0.75 ~ 1.72) | 0.546 |  | 1.00 (0.65 ~ 1.53) | 0.994 |  | 0.93 (0.60 ~ 1.43) | 0.733 |
| MHOO | 1.67 (1.24 ~ 2.25) | **<.001** |  | 1.72 (1.27 ~ 2.33) | **<.001** |  | 1.69 (1.24 ~ 2.29) | **<.001** |
| MUOO | 2.29 (1.73 ~ 3.02) | **<.001** |  | 2.29 (1.71 ~ 3.05) | **<.001** |  | 2.12 (1.55 ~ 2.90) | **<.001** |
| OR: Odds Ratio, CI: Confidence Interval | | | | | | | | |
| Model1: Crude | | | | | | | | |
| Model2: Adjust: Age, Sex, Marital status, Race, Education, Income | | | | | | | | |
| Model3: Adjust: Age, Sex, Marital status, Race, Education, Income, Smoke, Drink, Physical activity, Depression, Diabetes, Hyperlipidemia, Hypertension | | | | | | | | |

**Supplemental Tables S3.** Association of BMI-metabolic phenotypes with risks of incident OA after excluding participants with incident OA during the first wave of follow-up.

| Variables | Model1 | |  | Model2 | |  | Model3 | |
| --- | --- | --- | --- | --- | --- | --- | --- | --- |
|  | HR (95%CI) | *P* |  | HR (95%CI) | *P* |  | HR (95%CI) | *P* |
| MHO |  |  |  |  |  |  |  |  |
| MHNW | 1.00 (Reference) |  |  | 1.00 (Reference) |  |  | 1.00 (Reference) |  |
| MUNW | 0.89 (0.57 ~ 1.40) | 0.612 |  | 0.81 (0.51 ~ 1.28) | 0.370 |  | 0.79 (0.50 ~ 1.26) | 0.328 |
| MHOO | 1.46 (1.09 ~ 1.97) | **0.012** |  | 1.51 (1.12 ~ 2.03) | **0.007** |  | 1.51 (1.12 ~ 2.03) | **0.007** |
| MUOO | 1.76 (1.33 ~ 2.33) | **<.001** |  | 1.79 (1.35 ~ 2.37) | **<.001** |  | 1.75 (1.29 ~ 2.37) | **<.001** |
| HR: Hazard Ratio, CI: Confidence Interval | | | | | | | | |
| Model1: Crude | | | | | | | | |
| Model2: Adjust: Age, Sex, Marital status, Race, Education, Income | | | | | | | | |
| Model3: Adjust: Age, Sex, Marital status, Race, Education, Income, Smoke, Drink, Physical activity, Depression, Diabetes, Hyperlipidemia, Hypertension | | | | | | | | |
